# Factors associated with the relapse in Ponseti treated congenital clubfoot

**DOI:** 10.1186/s12891-022-05039-9

**Published:** 2022-01-26

**Authors:** Wei Hu, Baoyi Ke, Xiao Niansu, Sen Li, Cheng Li, Xingming Lai, Xinyu Huang

**Affiliations:** Department of Spine and Osteopathy Ward, Guilin Peoples’ Hospital, No 12 Wenming road, Guilin, 540021 Guangxi China

**Keywords:** Clubfoot, Relapse, Ponseti method

## Abstract

**Objectives:**

We retrospectively investigated the clinical materials to seek the factors that lead to relapse after using the Ponseti method.

**Methods:**

We retrospectively reviewed all children with congenital clubfoot treated with the Ponseti method in our hospital from June 2008 to June 2013. The data included the following factors: age, gender, initial Pinari score, number of casts, number of feet (unilateral or bilateral), age at the first casting, age of mother, tenotomy, walking age, and compliance with using bracing. All investigations were conducted in conformity with ethical standards. This study was approved by Guilin Peoples’ Hospital Ethics Committee.

**Results:**

In this study, there were 148 cases (164 ft) in total that underwent the Ponseti method, with the follow–up period at least 5 years. Of them, 64 children presented with left side, 58 with right side, and 26 with bilateral cases. This study included 75 males and 73 females; sex did not affect the outcomes. The mean age of the first casting was 2.50 ± 2.15 months. The average initial Pirani score was 4.98 ± 1.33, and the average number of casts was 5.71 ± 2.28 times. The mean age of mothers at birth was 25.81 ± 2.38 years old. The walking age of children was at a mean of 14.83 ± 1.18 months. Forty-nine cases could not tolerate using braces, namely the rate of noncompliance in this study was 33.1%. Tenotomy was performed on 113 ft (76.4%). The average follow–up period was 7.27 ± 1.29 years (from 5 to 10 years). The rate of relapse was 21.6% (32 cases) at the end of the follow-up. The rate of relapse in the noncompliance with using bracing group was significantly higher compared to the compliance group .

**Conclusion:**

The initial Pirani score, compliance with the foot abduction brace and the age at the first casting are three independent factors for relapse in clubfoot.

## Introduction

Congenital clubfoot is a common deformity of children, which consists of equinus, hindfoot varus, forefoot adductus and cavus [1]. The etiology of Congenital Clubfoot is largely unknown [2]. There is a consensus that the initial treatment of congenital clubfoot should be nonoperative [3, 4]. The Ponseti method was superior to surgery for treatment of clubfoot and achieved better long-term morphological, functional and radiological results [5]. The Ponseti method, which offers the safest, most effective, and least expensive correction of the majority of clubfeet, is now known as the golden standard to correct congenital clubfoot [6, 7]. Ponseti method is a effective technique in severe clubfoot as well as in common types [8]. However, relapses are common in severe clubfeet and are probably caused by the same pathology that initiated the deformity. The rate of recurrence after using the Ponseti method, occurring in up to 40% of patient [9]. The factors that relate to the recurrence are still debatable [10]. Therefore, we retrospectively investigated the clinical materials to seek the factors that lead to relapse after using the Ponseti method.

## Materials and methods

### Data collection

We retrospectively reviewed all children with congenital clubfoot treated with the Ponseti method in our hospital from June 2008 to June 2013. The data before treatment and during the follow-up period were recorded by two experienced authors of the Ponseti method and casting technique. The records included the following factors: age, gender, initial Pinari score, number of casts, number of feet (unilateral or bilateral), age at the first casting, age of mother, tenotomy, walking age, and compliance to brace using. This study was approved by the Ethics Committee Board of Guilin Peoples Hospital.

#### Inclusion criteria

1. We selected the clubfoot patients undergoing the Ponseti method with a minimum follow-up period of 5 years.
2. Untreated typical clubfoot.

#### Exclusion criteria

1. The patients with clubfoot secondary to arthrogryposis multiplex congenita or cerebral palsy, other than congenital clubfoot were excluded.
2. Clubfeet treated by surgery or non-Ponseti casting already were excluded.

### Casting technique

We used the Ponseti method and weekly casting to treat the deformity. The aim of the first casting was to correct the cavus. The castings that followed were to correct the midfoot inversion and heel varus. Supinating the forefoot to correct the malalignments was considered as the first step of the casting; thus, the cavus could be remedied. It is believed any attempt to correct the equinus before the correction of the heel varus and foot supination would result in a rocker bottom deformity. We reapplied a long leg cast weekly to keep the ankle in an externally rotated position until reaching 70 degrees. Percutaneous Achilles tenotomy was performed in the patients with equinus or with dorsiflexion of the ankle less than 10 degrees [11]. After this treatment, the last long leg cast was worn for 4 weeks.

### Judgment of compliance with foot abduction brace use

Once the deformity was corrected, the baby patients were instructed to use full-time bracing 23 h a day for 3 months (could only take off for a bath for less than 1 h), then altered from full-time to sleep time bracing (14–16 h a day) until 4 years old.

The toddlers of walking age were informed to use bracing 18 h a day for 3 months and moved to sleep time bracing (14–16 h a day) until 5 years old.

Since lacking of unanimous criteria to judgement of compliance with brace use [12], not wearing the brace for at least 75% of the number of hours prescribed above were defined as noncompliance [13].

### Define of relapse

#### Relapses in infant

The infant shows loss of foot abduction and/or of dorsiflexion correction with recurrence of adductus and cavus.

#### Relapses in toddlers

Supination of the forefoot, Varus of heel, the ankle range of motion and loss of passive dorsiflexion, any of the above happens is defined as relapse.

### Data analysis

After data collection being selected, the outcomes were analyzed independently by two authors (Li CH, Lai XM and Huang XY) who was blind to the study. Multivariable regression analysis was used to identify the independent factors for prediction of relapse.

### Statistical evaluation

We used SPSS version 18 (SPSS Inc., Chicago IL) for descriptive and statistical analysis. For comparing two independent means of variables in subgroups, we used the independent t-test. Nonparametric variables were assessed with the Fisher exact test and chi-square test. A *P* value of less than 0.05 was deemed to be significant.

## Result

In this study, there were 148 cases with 164 ft in total that underwent the Ponseti method. Of them, 64 children presented with left side, 58 with right side, and 26 with bilateral cases. This study included 75 males and 73 females; sex did not affect the outcomes (*P*=0.060). The mean age of the first casting was 2.50 ± 2.15 months. The average initial Pirani score was 4.98 ± 1.33, and the average number of casts was 5.71 ± 2.28 times. The mean age of mothers at birth was 25.81 ± 2.38 years old. The walking age of children was at a mean of 14.83 ± 1.18 months. Forty-nine cases could not tolerate using braces, namely the rate of noncompliance in this study was 33.1%. 113 ft (76.4%) were performed tenotomy by the same orthopedic surgeon (Baoyi Ke), who was accepted strict training before performing tenotomy (Table 1).

**Table 1:** Factors may be associated with relapse in clubfoot treated with Ponseti method

Variables	Relapse clubfeet	Nomal clubfeet	Statistical test	*P* value
Sex			3.548	0.060
male	11	64		
female	21	52		
Number of feet			3.895	0.143
left	12	52		
light	17	41		
bilateral	3	23		
Age at 1st casting (month)	3.62 ± 3.28	2.19 ± 1.61	2.377	0.023
Mother’s age	25.48 ± 2.60	25.91 ± 2.31	−1.003	0.317
Initial Pirani score	5.56 ± 0.47	4.82 ± 1.45	4.678	0.000
Number of casts	7.15 ± 3.05	5.20 ± 1.67	8.450	0.000
Tenotomy			2.810	0.094
Yes	28	85		
No	4	31		
Walking age	14.79 ± 1.10	14.92 ± 1.24	−0.503	0.616
Compliance to brace using			48.453	0.000
yes	5	94		
no	27	22		

The average follow–up period was 7.27 ± 1.29 years (from 5 to 10 years). The rate of relapse was 21.6% (32 cases) at the end of the follow-up. The rate of relapse in the noncompliance with using bracing group was significantly higher compared to the compliance group (55.10% versus 5.15%, *P* = 0.000).

Multivariable regression analysis showed the age of the first casting, initial Pirani score and compliance with using bracing were three independent factors to predict relapse after treatment with the Ponseti method. While, there was no correlation between gender, number of casts, side of involvement, tenotomy, age of mother and walking age (Table 2).

**Table 2:** Outcome of multivariable analysis: factors may be associated with relapse

variable	B	S.E.	Wald	df	Sig.	OR
Age at 1st casting	.475	.178	7.113	1	.008	1.609
Initial Pirani score	1.343	.535	6.303	1	.012	3.832
Compliance to brace using	−3.667	.657	31.173	1	.000	.026

## Discussion

Nowadays, the Ponseti method is the first choice for congenital clubfoot, which consists of manipulation, casting, and Achilles tenotomy. This conservative method significantly reduces the complications of the extended surgery, such as stiffness and arthritis of the foot in adulthood. Previous studies reported relapse rates up to 40% [9]. However, a common question that is usually raised is, “What factors are associated with relapse?” This study analyzed a combination of clinical factors for their association with relapse after treatment with the Ponseti method. One of the advantages of multivariable regression analysis is that it can identify the most significant factors that doctors need to consider when making decisions. This work has retrospectively reviewed 164 ft in 148 cases with an average 7.27 years of follow-up. It shows the result that the age at the first casting, high Pirani scores and noncompliance with foot abduction bracing are the main risk factors for the relapse of congenital clubfoot after treatment with the Ponseti method. However, no correlation between gender, number of casts, side of involvement, tenotomy, age of mother and walking age was found. There is a previous study shown the similar outcome [14]: male and female patients and patients with unilateral or bilateral involvement performed equally well, and no limitations in sport performance or activity could be observed.

Foot abduction bracing appears to be important in maintaining the correction after the Ponseti method. Noncompliance with the foot abduction brace was considered the leading influential factor for relapse in congenital clubfoot corrected by the Ponseti method [15]. Several main reasons for noncompliance with the foot abduction brace have been identified. First, there are no defined criteria for judgment of compliance with the foot abduction brace [12]. Ambiguous brace protocols might result in different clinical outcomes. The second reason is that the children may no longer be compliant with the brace, especially after the age of 1.5 years old. During the time they grow up, they tend to sleep less than before; therefore, less brace wearing occurs, because children are unlikely to tolerate the bar-connected brace when they are awake. Moreover, the parents have insufficient understanding of the importance of the brace. Once the noncompliance occurs, some parents may not strictly adhere to the principles of using the brace, even abandoning its use. In some cases, the casting was removed at home the day before the cast changing for the purpose of reducing the amount of time in the hospital. A great amount of correction is lost, because the foot is out of the cast for a few hours or even all night. In fact, the cast should not be removed for more than an hour before the new cast is applied. In our study, we find that the relapse rate is 21%, the relapse rates of noncompliance were 5.4 times greater than that of compliance and there is a positive correlation between noncompliance and relapse, which indicates that relapse could be associated with an insufficient time of wearing the foot abduction brace. So, it is extremely crucial to take every opportunity to make the parents aware of the meaning of wearing the brace. The doctors should repeatedly emphasize this every time when changing the casting every week to make the guardians understand that the bracing is as equally important as the Ponseti casting and tenotomy.

The Pirani scoring system is reliable, quick, and easy to use. So, it is widely used for classifying and predicting the treatment of clubfoot. A study showed the initial Pirani score’s influence on the number of Ponseti casts [16]. Whether the initial Pirani score influences the relapse of clubfoot still remains controversial. Several studies revealed that the role of the Pirani score decreased in older children [17]. A new study reported [18] that Pirani scores are markedly related with recurrence in patients with severe and very severe clubfoot. Our study reveals that the initial Pirani score is an independent factor for relapse in clubfoot as well as compliance to the foot abduction brace and the age at the first casting. Therefore, it is necessary to inform the parents whose children have a high initial Pirani score to closely follow-up, thus, any recurrence can be identified earlier.

There is a consensus that the Ponseti method should be performed as soon as possible; however, there is another debatable study reporting that age at the beginning of treatment does not influence the final outcome [19]. Therefore, whether the age at first casting influences the effect of correction and the correlation of the relapse is still controversial. The pathology has shown the ligaments in the newborn are full of bundles of collagen fibers, which allows the ligaments to be stretched. This makes the manipulation to correct the deformity feasible. As the child grows up, the collagen synthesis in the ligaments, tendons, and muscles may persist until the child is 3 or 4 years of age and might be a cause of relapse. Therefore, treatment should be performed as quickly as possible to take advantage of the favorable elasticity of the tissues, such as the ligaments, joint capsule and tendons of the newborn. It is suggested that treatment should begin in the first week of life. However, the skin of the newborn is too tender to break. Once there is a break, a sore occurs and the casting should be taken off for at least 1 week for the sore to heal. So, with all the newborns in this study, we started the treatment at least older than 1 month of age to avoid the sores, because the condition of the skin is firmer after 1 month.

## Conclusion

The initial Pirani score, compliance with the foot abduction brace and the age at the first casting are three independent factors for relapse in clubfoot.

### Study limitations

There are limitations to this study. First, it is a single-center retrospective rather than a randomized controlled trial, namely without Class I evidence. Second, the study reveals that the initial Pirani score is an independent factor for relapse in clubfoot. But what score is considered high enough to inform the parents to closely follow-up, the study does not do further research to answer the question. Moreover, the sample size we collected may not have been large enough to influence the results such that potential significant differences were not found. Thus, it is possible that a longer multicenter follow-up study might be necessary to predict the long-term outcome of this specific treatment option.

All methods were performed in accordance with the relevant guidelines and regulations.

## Data Availability

All the data needed to achieve the conclusion are presented in the paper.
